# Sentiments of Individuals with Interstitial Cystitis/Bladder Pain Syndrome Toward Pentosan Polysulfate Sodium: Infodemiology Study

**DOI:** 10.2196/54209

**Published:** 2025-01-17

**Authors:** Yulin Hswen, Qiuyuan Qin, Pressley Smith, Alison Swierczynski, Stuart Bauer, Erika Ladson, Amanda Leigh Garrett, Catherine A Brownstein

**Affiliations:** 1 Department of Epidemiology and Biostatistics, University of California San Francisco, San Francisco, CA, United States; 2 Department of Public Health and Sciences, University of Rochester, Rochester, NY, United States; 3 Division of Genetics and Genomics, Boston Children's Hospital, Boston, MA, United States; 4 Department of Urology, Boston Children's Hospital, Boston, MA, United States; 5 Department of Surgery, Harvard Medical School, Boston, MA, United States; 6 Inspire, Arlington, VA, United States; 7 Department of Pediatrics, Harvard Medical School, Boston, MA, United States

**Keywords:** interstitial cystitis, IC, painful bladder syndrome, bladder pain syndrome, BPS, social media, social network, pain, treatment, chronic condition, chronic disease, chronic illness, Elmiron, pentosan polysulfate sodium, PPS, internet forum

## Abstract

**Background:**

Interstitial cystitis/bladder pain syndrome (IC/BPS) is a multifactorial, chronic syndrome involving urinary frequency, urgency, and bladder discomfort. These IC/BPS symptoms can significantly impact individuals’ quality of life, affecting their mental, physical, sexual, and financial well-being. Individuals sometimes rely on peer-to-peer support to understand the disease and find methods of alleviating symptoms. The only US Food and Drug Administration–approved medication to treat IC/BPS is pentosan polysulfate sodium (PPS). However, ocular pigmentary maculopathy has been described in some individuals, with greater severity associated with prolonged PPS exposure.

**Objective:**

While prior research has separately assessed the benefits and side effects of PPS, this study sought to identify (1) sentiments of individuals with IC/BPS toward PPS and (2) topics discussed by individuals with IC/BPS in conjunction with PPS through use of an internet peer-to-peer forum.

**Methods:**

Data were collected from Inspire—an anonymous web-based health community where individuals gather by condition to find support and information. Sentiment analysis and percentages of negative, positive, and neutral sentiment for PPS discussions encompassing each topic was conducted using VADER (Valence Aware Dictionary for Sentiment Reasoning). Topic modeling was conducted using latent Dirichlet allocation. Words with the highest probability were ranked to categorize each topic, and authors manually investigated and labeled discussions.

**Results:**

There were 354 forum posts related to PPS. Topic modeling with latent Dirichlet allocation revealed 5 topic categories: “ineffectiveness or discontinued use,” “alternative treatments,” “personal treatment suggestions based on experience,” “severe side effects,” and “risk of long-term use.” Topics related to “severe side effects” and “risk of long-term use” garnered less discussion, with the former also having the lowest positive sentiment (4.28, 14.29%). The topic “ineffectiveness or discontinued use” was most frequently discussed. This topic also had the highest percentage of negative posts (52/152, 34.21%). However, the average compound score was within the neutral compound score range (−0.094, SD 0.625). In addition, forum data highlighted individuals’ acknowledgment of the efficacy of PPS in improving their quality of life, with statements such as “saved my sanity” being representative. The overall compound individuals’ sentiment toward PPS was −0.083, split across 32.49% (115/354) negative, 22.03% (78/354) positive, and 45.48% (161/354) neutral sentiment categories.

**Conclusions:**

The overall authentic sentiment toward PPS is broad but balances to neutral. This neutral sentiment suggests that while some individuals express concerns about the side effects and long-term risks associated with PPS, others appreciate its positive impact on their quality of life. This research confirms that individuals with IC/BPS actively engage with health forums like Inspire to seek information, share their experiences, and explore different treatment options. As IC/BPS remains a complex syndrome, this study highlights the value of patient-led discussions in informing treatment decisions. Furthermore, these findings suggest that health care providers might benefit from considering the insights shared on peer-to-peer forums to better understand individual preferences, concerns, and expectations.

## Introduction

### Interstitial Cystitis/Bladder Pain Syndrome and Current Treatment

Interstitial cystitis/bladder pain syndrome (IC/BPS) is a multifactorial chronic syndrome that affects the urinary bladder [1]. The condition is characterized by urinary frequency, urgency, and bladder pain [1], involving both women and men [2], that impact their emotional, psychological, and social well-being [1]. The etiology of IC/BPS remains elusive; the disease is diagnosed not with a single test but a combination of multiple investigations to rule out other diagnoses [3]. Many individuals with IC/BPS present heterogeneous syndromes from several causes [4] suggesting it should be classified into different subtypes [5]. Recent investigations of genetics and biomarkers have begun to uncover the molecular mechanisms of IC/BPS [4,6]. First-degree relatives of women with IC/BPS have a greater prevalence of the disease than women without affected relatives, indicating a genetic component [7,8]. Twin studies have shown a greater concordance of IC/BPS among monozygotic than dizygotic twins [7,9]. The Golgi-localized adenosine triphosphatase gene *ATP2C1* has also been identified as having increased rare variant burden in an IC/BPS cohort (OR [odds ratio]=6.76, 95% CI 1.1-75.9) [6] and is a candidate gene.

### Pentosan Polysulfate Sodium–Associated Pigmentary Maculopathy

Currently, the only US Food and Drug Administration (FDA)–approved treatment is oral pentosan polysulfate sodium (PPS). Use of PPS has been demonstrated to improve symptoms [10], although recent studies link PPS to a unique presentation of pigmentary maculopathy [11]. Pigmentary maculopathy can produce moderate visual impairment, macular hyperpigmented spots, yellow-orange deposits, and patchy retinal pigment epithelium atrophy [12]. The first study detailed 6 individuals over 2 years who developed new visual symptoms after exposure to PPS [11]. Several additional studies have replicated the association between PPS use and pigmentary maculopathy [13-16].

### Support Groups and the Inspire Platform

Patients and caregivers often join web-based health communities to find support for incurable or rare diseases. Through interpersonal exchanges, patients and caregivers can develop an understanding of their illnesses. Social Comparison Theory postulates that social behaviors could be predicted on the assumption that individuals seek the maintenance of normalcy and accuracy in their world by using opinions of others to evaluate their thinking and feeling [17]. The benefits of support groups can be explained by social support proximal factors, for example, attachment, group membership, and collective identity [18]. Secure adult attachment is described as having comfort, intimacy, and trust such that another person can provide support, empathy, and care. Thus, individuals with chronic illnesses seek comfort from others who can provide this type of attachment [18]. There is also evidence that attachment security benefits individuals with health-related distress, as it was found to be negatively associated with depression among individuals with chronic pain. Therefore, illness support groups can provide security and a positive effect on individuals [18]. Many forums allow users to maintain anonymity, which can encourage more honest and candid discussions, especially about sensitive topics, while being easily accessible to anyone with internet access.

Group membership also describes the evolution of humans having an automatic tendency to categorize themselves and others as in-group and out-group based on similar characteristics, for example, having the same chronic illness [18]. Individuals with chronic illnesses sometimes deviate from social norms, that may impact their physical appearance and actions and can lead to stigmatization, discrimination, and social exclusion, threatening a sense of belonging. Due to these effects, individuals look toward in-group membership, leading to beneficial cohesion [18].

The perceived benefits of participating in support communities include meeting and befriending others with the same condition and similar experiences, learning about the disease and related treatments, giving and receiving emotional support to group members, and accountability for reaching health-related goals [19-21]. Digital communities often serve as spaces where users collaborate to find solutions to common challenges, drawing on the collective wisdom of the group [22].

The Inspire platform is the world’s largest and fastest-growing health community designed as an environment for individuals to feel comfortable sharing personal experiences and sensitive health information on an anonymous forum. More than 1o million annual users on the Inspire platform have joined hundreds of disease-specific community groups with patients and caregivers with similar diseases [23]. The Inspire platform maintains anonymity while allowing individuals to obtain information about, and participate in, clinical trials and research efforts [23]. The Interstitial Cystitis Association community on Inspire has over 40,000 members and is the most active IC/BPS forum [23].

Web-based communities provide a rich source of information for health research [24]. For example, monitoring web-based social networks has been an effective tool for identifying adverse events [25] and barriers to accessing health care [26]. The use of web-based social networks for disease surveillance is well documented [27-30]. Notably, data from web-based networks has also been used to obtain a deeper understanding of patient sentiment [31]. Therefore, this study sought to identify (1) sentiments of individuals with IC/BPS toward PPS and (2) topics discussed by individuals with IC/BPS in conjunction with PPS through use of an internet peer-to-peer forum.

## Methods

### Study Design and Data Sources

To examine both the sentiment and the topics discussed concerning PPS, the Inspire forum for individuals with IC/BPS was used to derive quantifiable data points. We used the IC/BPS discussion forum posts and replies from the United States from January 12, 2019, to November 30, 2022. In our analytical sample, 3232 patient forum posts were collected from the Inspire forum, and 354 discussed PPS. The data corpus was created to gain insight into the triggers or factors flaring or worsening IC/BPS symptomology. As such, specific text terms and razor tags were used to bring these topics to the surface: flare-, flair-, worse-, increase-, more, trigger-, caus-, inducing, induce-, producing, produce-, factor, factors, provok-, activat-, exacerbate-, irritat-, hypersensitive-, sensiti-, react-, risky, acute, #flare, #riskfactor, #environmentalfactor, #cause, #causality, #hypersensitivity, and #sensitization. Texts were analyzed for specific discussions regarding PPS and other concerns of individuals with IC/BPS. Conversations and discussion posts were limited to sentences with the term PPS and the trade name for PPS. These sentences were used for further analysis alongside the previous sentence relative to the usage of “PPS” and the following sentence relative to the usage of “PPS”.

All analyses used this definition of PPS-related discussions and were conducted using Python version 3.6 (Python Software Foundation). In addition, a combination of topic modeling and sentiment analysis was used in the retroactive investigation.

### Topic Modeling

Topic modeling is statistical modeling for discovering abstract “topics” in a text collection. Latent Dirichlet allocation (LDA) is a statistical topic model used to discover and classify text to a particular collection. LDA builds a topic per document model and words per topic model, creating Dirichlet distributions. Each topic has a probability of generating different words in the text. Topics are surfaced based on the likelihood of word co-occurrence and the probability of words making up each topic.

Before using LDA for topic modeling, raw data were preprocessed (lower cased, removing URL links, nonletter characters, and abbreviations) and tokenized (divided into sets) by returning a list of strings for each post to prepare for analysis using the GenSim package in Python [32]. Furthermore, bigrams were used on the tokenized data to combine frequently occurring words to analyze topics. Bigrams were also implemented on the tokenized data to combine the words that occurred together frequently. Common stop words such as “the,” “a,” “an,” “in,” and so on, were filtered out using the Natural Language Toolkit in Python, which contains a list of stop words stored in the English language [33]. The spacy package was used to lemmatize words (reducing words to their root word, eg, “running” and “ran” can be reduced to “run”) and ensure the thorough removal of stop words. The words “PPS” (and the associated trade name) were also excluded from the LDA analysis because the data were filtered explicitly for discussions about “PPS.” For the intertopic distance map, multidimension scaling is used for the visualization of the topics in a 2D space with the 2 principal components, prinicipal component 1 and principal component 2. We used coherence score to select the potential numbers of topics for LDA model. Coherence score measures the consistency, clarity, and relevance of each topic in LDA modeling [34,35]. We then selected the maximum number of topics that could be produced without overlapping the intertopical distance map. We also used perplexity score to examine the model performance. Perplexity score measures the predication performance of a LDA model on held-out documents [36,37]. The size of the topic represented by circles, is proportional to the proportion of posts about PPS by individuals on Inspire. Topics having more words in common are represented closer together, thus the distance between the topics is related to how different the topics are in the posts.

After preprocessing and tokenizing the data, each forum post was categorized to the LDA topic with the highest proportion to identify hidden topics within LDA modeling. Words with the highest probably were ranked for each topic. Finally, topics were manually labeled to determine the overarching theme for each topic (AS, EL, CAB, and ALG).

### Sentiment Analysis

Sentiment analysis was used to computationally identify and categorize opinions expressed on the IC/BPS Inspire forum to find the emotional tone behind PPS-associated text. Sentiment analysis was conducted using the Valence Aware Dictionary for Sentiment Reasoning (VADER). VADER is a lexicon and rule-based sentiment analysis tool to identify the sentiment attributed to a topic of interest. It is also considered the standard for analyzing sentences to determine the sentiment of a piece of literature.

The results generated by VADER include a dictionary of 4 percentage scores classified as negative, neutral, positive, and compound. The negative, neutral, and positive scores are the relative percentages of negative, neutral, and positive sentiments surrounding the topic discussion. The compound score is a continuous score ranging from −1 (most negative) to +1 (most positive). Definitions most commonly used for compound scores are: −1.0 to −0.5 (a negative score), −0.5 to +0.5 (a neutral score), and +0.5 to +1.0 (a positive score). Sentiment analysis was conducted on all PPS-related discussions and on all topics determined by LDA topic.

### Ethical Considerations

Inspire community members may anonymously engage with others through public discussion posts in the forum. The participants agree to have their posts or discussion comments used for research purposes upon registering for Inspire. Deidentified community posts, with all personally identifiable information removed, are available for secondary data analysis. Individuals were not compensated for participating in the forum. This study was approved by the Boston Children’s Hospital Institutional Review Board (IRB-P00040767).

## Results

### Overview

Results are based on the 354 posts that related to PPS. Examples of forum posts with sentiment categorization by VADER are listed in Table 1.

**Table 1.** Select interstitial cystitis/bladder pain syndrome forum posts describing their experiences with pentosan polysulfate sodium. Posts with positive, negative, and neutral sentiment are shown.

Example notable quotes	Sentiment categorization by VADER^a^
“IC is not just an inflamed bladder. It is a disease with no known cause or cure. It is a lifetime of pain and misery. I know I have had it for over 20 years now. IC causes the lining of your bladder to shed each time you urinate. It is so painful it feels like lava coming through your thighs.”	Overall neutral sentiment toward topic 1 (ineffectiveness or discontinuing use)
“Interstitial Cystitis is insidious, terribly painful, and has been a direct causality of what I can and could not do throughout the entire duration of my lifetime.”	Overall negative sentiment toward topic 3 (personal treatment suggestions based on the experience)
“You must be patient and not give up hope, be willing to try different remedies as not one thing works for everyone. Relief can be fleeting and not every day since the condition is chronic.”	Overall positive sentiment toward topic 3 (personal treatment suggestions based on the experience)
“…with all the class action suits, I got scared and stopped taking [...] I have had several flare-ups since I stopped and no quality of life, as someone else said. I think eyesight is a big side effect to consider, but the alternative is pain [...] still deciding if I should go back on it?”	Overall neutral sentiment toward topic 1 (ineffectiveness or discontinuing use)
“[...] actually would give me a reasonable baseline of normal, but I have chosen to go off of it due to the new vision concerns. I am praying that they will determine the vision issue to be very low risk, and I will consider going back on because it is really what works best for me.”	Overall positive sentiment toward topic 2 (alternative treatment)
“I foolishly went off [...] in January because of the possible retinal eye damage and of course the high cost. Really big mistake. I’m back on [...] and hoping that it doesn’t take 3‐4 months for it to kick in again.”	Overall negative sentiment toward topic 3 (personal treatment suggestions based on the experience)
“a lifesaver because [they] also [were] in severe pain, burning, etc, and running to the bathroom. After [...], [they] had no pain, [they] could eat anything [they] wanted.”	Overall negative sentiment toward topic 5 (risk of long-term use)
“I am so glad to find someone besides myself that isn’t stopping [...] I was suicidal before [...] went to work on me. The fear of returning to the hot mess I was before taking [...] terrifies me. I am comforted to find out I am not the only one sticking with it.”	Overall negative sentiment toward topic 5 (risk of long-term use)
“[...] is garbage and has terrible side effects.”	Overall negative sentiment toward topic 1 (ineffectiveness or discontinuing use)
“antihistamines, amitriptyline, [...], and an ultra-strict diet seem to help for a few days, and then I am right back to where I started.”	Overall negative sentiment toward topic 1 (ineffectiveness or discontinuing use)

a VADER: Valence Aware Dictionary for Sentiment Reasoning.

In Table 1, all posts were analyzed using VADER for sentiment categorization. To determine sentiment, VADER begins by splitting the text into individual words. It then assigns each word a score to indicate whether it is positive or negative. Using these predefined scores, VADER computes an overall sentiment score for the entire text. VADER also accounts for sentiment intensity (eg, capitalization and punctuation) and looks for modifiers that might alter the meaning of nearby words.

### LDA Modeling Output

**Figure 1.**
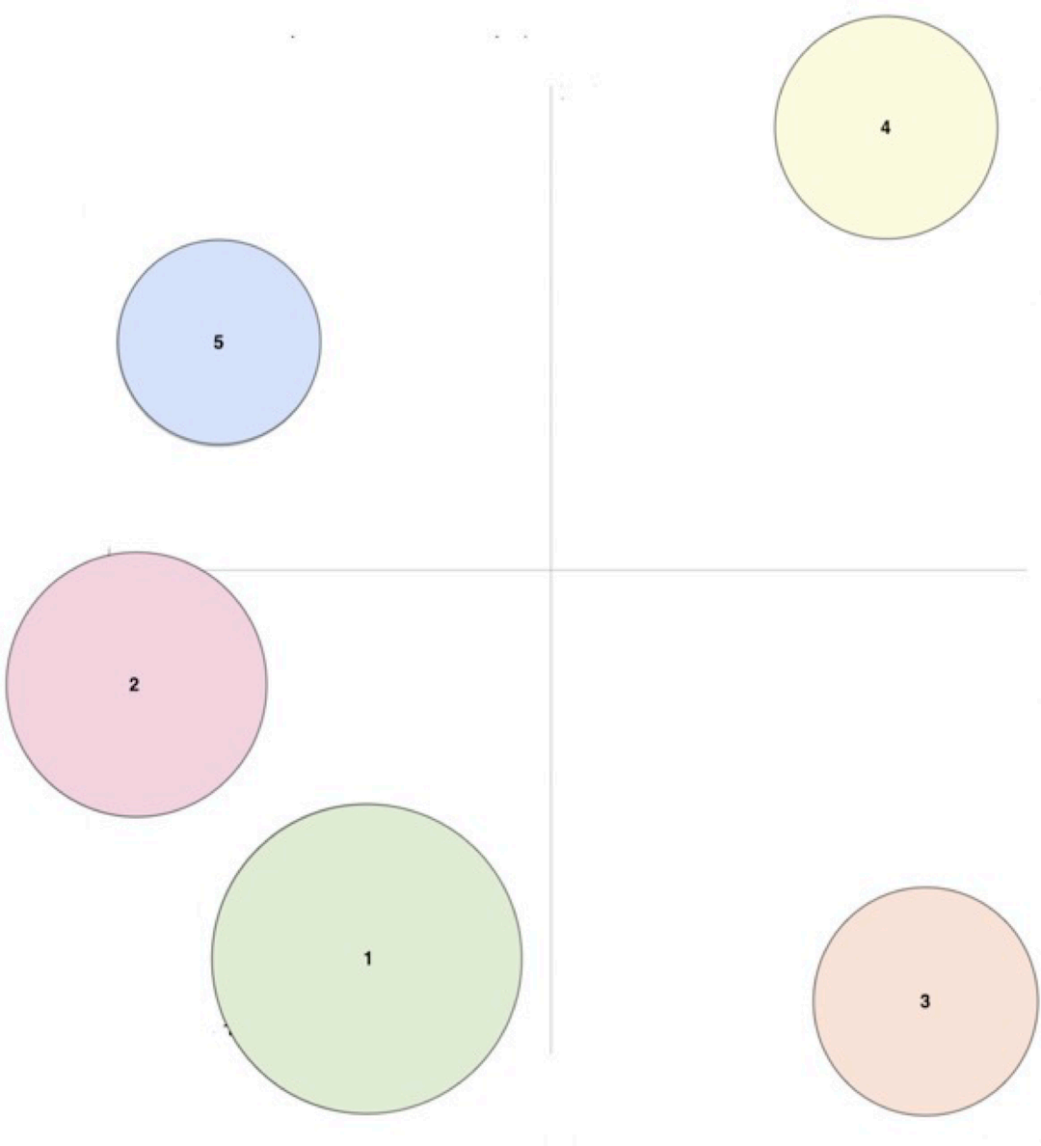
Display of intertopic distance and marginal distribution of the 5 topics. Instead of representing the divergence of topics as a phylogentic tree in 1D, this map allows visualization of how topics relate in 2D space–based principal components. Intertopic distance map measures the distance between topics. Similar topics are closer to each other, while very different topics are farther apart. The size of the circle represents the number of words in each topic; larger circles mean more words belong to that topic.

LDA topic modeling revealed 5 topics, the minimum number without overlap. Figure 1 displays the intertopic distance map, the area of the topic circles proportional to the number of words that belong to each topic. The distance between topics represents the degree of difference between each. Topics having more words in common are closer together. The greatest proportion of discussions was topic 1 (152/354), then topic 2 (92/354), topic 3 (56/354), topic 4 (28/354), and topic 5 (26/354). In Figure 1, topics 1, 2, and 5 were more closely clustered than topics 3 and 4, indicating they were most dissimilar to topics 1, 2, and 5. The highest probability of co-occurring words making up each LDA topic is shown in Table 2. Using the co-occurence of words and manual investigation, Topic 1 was identified as “ineffectiveness or discontinued use,” topic 2 as “alternative treatments,” topic 3 as “personal treatment suggestions based on experience,” topic 4 as “severe side effects,” and topic 5 as “risk of long-term use.”

**Table 2.** Latent Dirichlet allocation word occurrence probability for pentosan polysulfate sodium–related discussions in the interstitial cystitis/bladder pain syndrome forum.

Topic 1: ineffectiveness or discontinued use (152/354, 42.9%)		Topic 2: alternative treatments (92/354, 26%)		Topic 3: personal treatment suggestions based on the experience (56/354, 15.8%)		Topic 4: severe side effects (28/354, 7.9%)		Topic 5: risk of long-term use (26/354, 7.3%)	
Probability	Word	Probability	Word	Probability	Word	Probability	Word	Probability	Word
.08	Take	.08	Go	.12	Eye	.05	Try	.05	Night
.08	Year	.08	Also	.09	Damage	.04	Bladder	.04	Problem
.04	Stop	.07	Pain	.07	Cause	.04	Start	.04	Really
.04	Flare	.04	Help	.06	Retinal	.04	Have Been	.04	Think
.03	IC	.04	Month	.05	See	.03	Back	.03	Pigmentary
.03	Time	.03	Day	.04	Issue	.03	Continue	.03	Vision
.03	Work	.02	Capsule	.04	Hear	.03	Make	.03	Maculopathy
.03	Find	.02	Get	.03	Vision	.03	Still	.03	Macular degeneration
.02	Thing	.02	Med	.03	Retina	.03	Medication	.02	End
.02	Know	.02	Diet	.03	Take	.03	Burn	.02	Cost
.02	*Help*	.02	Bladder	.03	Ask	.02	Due	.02	Symptom
.02	People	.02	Give	.03	Drug	.02	Want	.02	Many
.02	Try	.02	Back	.03	Condition	.02	Give	.02	Much
.02	Dr	.01	Week	.02	Doctor	.02	Ever	.02	Risk
.02	Hope	.01	Mg	.02	Long	.02	Instillation	.02	Course
.02	Lot	.01	Seem	.02	Hair	.02	Fall	.02	Patient
.02	Life	.01	Urologist	.02	Light	.02	Free	.02	Hour
.02	Month	.01	Time	.01	Specific	.02	Develop	.02	Concern
.01	Possible	.01	Food	.01	Term	.02	Little	.02	Hydroxyzine
.01	Day	.01	Eat	.01	Stop	.02	Pill	.02	Helpful

In Table 2, the LDA modeling method is used to reveal the main topics and how they are distributed across a dataset (ie, the IC/BPS forum). Five main topics were identified. The proportion, number of posts discussing each topic, and manual topic labels for each LDA topic are shown.

### Sentiment and Topic Analysis

The overall sentiment scores for each topic are presented in Table 3. Overall, the VADER compound sentiment of discussions about PPS was −0.083, (negative sentiment 32.49% [115/354], positive 22.03% [78/354], and neutral 45.48% [161/354]).

**Table 3.** Sentiment scores for discussions surrounding pentosan polysulfate sodium (n=354). Posts were stratified by the identified topics from the latent Dirichlet allocation analysis.

Topic number and label	Average compound score (SD)	Negative, n (%)	Positive, n (%)	Neutral, n (%)
Total (n=354)	−0.083 (0.603)	115 (32.49)	78 (22.03)	161 (45.48)
Topic 1: ineffectiveness or discontinuing use (n=152)	−0.094 (0.625)	52 (34.21)	35 (23.03)	65 (42.76)
Topic 2: alternative treatments (n=92)	−0.082 (0.620)	31 (33.7)	23 (25)	38 (41.3)
Topic 3: personal treatment suggestions based on the experience (n=56)	−0.004 (0.573)	15 (26.79)	12 (21.43)	29 (51.79)
Topic 4: severe side effects (n=28)	−0.162 (0.523)	9 (32.14)	4 (14.29)	15 (53.57)
Topic 5: risk of long-term use (n=26)	−0.119 (0.581)	8 (30.77)	4 (15.38)	14 (53.85)

In Table 3, each topic number represents a specific theme that emerged during the analysis of the text corpus. The average compound score indicates whether the overall tone of discussions for each topic is positive, negative, or neutral. The number and percentage of scored negative, postitive, and neutral posts is shown. By comparing sentiment across these topic numbers, we can gain insights into how different aspects of PPS are perceived within the discussions.

Topic 1 identified by the LDA topic modeling was manually labeled “ineffectiveness” based on the total posts about PPS on the IC/BPS Inspire forum using its relative size in Figure 1 and number of posts in Table 3. In Topic 1, the highest probability of co-occurring and relevant words compromising this topic were: take (.08), year (.08), stop (.04), and flare (.04). Its distance in relationship to topics 2 (alternative treatments) and 5 (risk of long-term use), seen in Figure 1, indicates a higher degree of similarity based on the types of words they have in common. Topics 4 (severe side effects) and 3 (personal treatment suggestions based on experience) were furthest from topic 1. shows the overall compound scores, and percent negative, positive, and neutral for the entire dataset and for each topic of PPS posts by individuals from Inspire. Topic 1 had the highest percentage of negative posts with 34.21% (52/152), 23.03% (35/152) positive sentiment posts, while the percent of neutral sentiment was 42.76% (65/152). However, despite having the most percent negative posts compared with any other topic, the average compound score was −0.094 which falls within the neutral compound score range of −0.50 to +0.50.

Topic 2 was identified by LDA modeling and manually labeled as “alternative treatments.” This topic had the most overlap of words in common with topic 1 Figure 1. Topic 2 was the second largest topic with 26% (92/354) of the total posts about PPS on the IC/BPS Inspire forum. The highest probability of co-occurring words most relevant terms to the topic was: pain (.07), help (.04), diet (.02), urologist (.01), time (.01). Moreover, it had the second-largest proportion of discussions on the IC/BPS Inspire forum and the second most posts associated with the topic; note its relative size (Figure 1). Compared with the other 4 topics, topic 2 had the second-highest percentage of negative posts at 33.7% (31/92). However, it also had the highest percentage positive sentiment score (23/92, 25%) and percentage of neutral (38/92, 41.3%). The overall compound score in topic 2 was −0.082, the second most negative, falling within the neutral compound score range of −0.50 to +0.50.

Topic 3 was identified by LDA modeling and manually labeled as “personal treatment suggestions based on experience.” It was the third largest topic with 15.8% (56/354) about PPS on the IC/BPS Inspire forum. The highest probability of co-occurring words most relevant to the topic was: eye (.12), damage (.09, retinal (.06), vision (.03), retina (.03), and doctor (.02). Compared with the other 4 topics, topic 3 had the least negative compound sentiment (−0.004) and the least percent of negative (15/26, 26.79%), positive (12/56, 21.43%), and (29/56, 51.79%) neutral posts. This is reflective of the distance of topic 3 in the intertopic distance map whereby it is further from to topic 2.

Topic 4 was identified by the LDA modeling and manually labeled as “severe side effects.” It was the second smallest topic with 7.9% (28/354) of the total posts about PPS on the IC/BPS Inspire forum. The highest probability of co-occurring words most relevant to the topic was bladder (.04), medication (.03), burn (.02), and instillation (.02). It had the highest intertopic distance compared with the topics 2, 1, and 3, making it the least similar topic based on word commonality. Topic 4 had the most negative compound score, −0.162 with 32.14% (9/28) negative, 14.29% (4/28) positive, and 53.57% (15/28) neutral posts.

Topic 5 was identified by the LDA modeling and manually labeled as “risk of long-term use.” It was the smallest topic with 7.3% (26/354) about PPS on the IC/BPS Inspire forum. The highest probability of co-occurring words most relevant to the topic was: night (.05), problem (.04), pigmentary (.03), vision (.03), maculopathy (.03), and macular degeneration (.03). The average compound score for was −.119 (SD 0.581) which falls within the neutral compound range of −0.50 to +0.50. It had the highest percentage of neutral sentiment posts (14/26, 53.85%), compared with the other topics.

## Discussion

### Principal Findings

PPS is widely prescribed to relieve IC/BPS symptoms [1]; it is the only FDA-approved oral medication available for symptom alleviation. However, PPS has been linked to a unique presentation of pigmentary maculopathy [16]. This potential side-effect has created a need for individuals exposed to PPS to be consistently monitored by an optometrist for early detection [11,13].

Although previous studies have assessed the benefits and side effects of PPS, they have done so separately without capturing individuals’ thoughts on if or why they are willing to assume the side effects for the relief of pain. This study provides information as to the sentiment of the IC/BPS community toward PPS, which was determined to be neutral, with 5 topics discussed in the forum all producing a neutral compound score.

Studying sentiment toward medications offers several important benefits, beginning with improved patient-centered care that aligns with individual preferences, concerns, and quality of life. An understanding of patient sentiment may assist health care professionals in assessing whether the perceived benefits of a medication outweigh the risks from the patient’s perspective. As the data here indicate that patient sentiment is neither strongly for nor against PPS, patients may be less influenced by emotional or external biases, making it easier to assess the medication’s effects based on objective outcomes rather than preconceived notions. This can inform shared decision-making and guide personalized treatment options. It is arguable that understanding patient’s sentiment toward their medication could reduce anxiety and improve trust, both of which are related to treatment adherence [38].

As a whole, the forum data describe a willingness among some individuals to risk the potential side effects associated with PPS due to its effectiveness at relieving pain. Multiple posts indicate PPS, “saved [their] sanity” or, “changed [their] life” (Table 1). However, the proportion of negative sentiment is close to one-third (115/354, 32.49%), with positive sentiment taking up a proportion of just over one-fifth (78/354, 22.03%) and neutral just under one half (161/354, 45.48%). The mean compound sentiment score in the forum is −0.083 (SD 0.603), indicating an overall neutral sentiment.

Topics 4 (severe side effects) and 5 (risk of long-term use), were the least discussed. While topic 4 did not surface as often in the LDA model and had a lower discussion frequency, it did have the lowest positive sentiment. Topic 5 had the highest neutral sentiment of the topics.

### Limitations

Some drawbacks of using web-based health communities such as Inspire include difficulty controlling the quality of information shared on such sites. Uncertain and unknowable generalizability of these results is another limitation. Other potential drawbacks include privacy risks to patients and caregivers utilizing the platform, as “data intruders” could lead to discrimination by employers, insurance companies, family, and friends. Although there are policies, such as the Genetic Information Nondiscrimination Act, which prohibits genetic information discrimination in employment, privacy is still a risk that must be considered [20] and individuals may be unwilling to speak freely.

VADER uses a lexicon-based approach system. In such an approach, while broader term coverage is made available, words have a finite assignment to sentiment orientation, which does not consider figures of speech or negations, as mentioned above [39]. LDA topic modeling limitations include the number of topics that can be generated and the inability to depict correlations [40,41]. Topics are not developed over time, and the modeling assumes that words are exchangeable and that sentence structure is not modeled.

Confounding factors, such as current symptoms and overall IC/BPS management, should be included in future studies to further the understanding of the underlying determinants of sentiment toward PPS. Comparitive attitudes toward different IC/BPS treatments should also be explored to assess varying disease management strategies.

### Conclusions

Individuals use health forums such as Inspire to learn more about their disease, medications, and treatments. Therefore, this study aimed to use forum posts from Inspire to investigate individuals with IC/BPS’ overall sentiment toward PPS. Using VADER, sentiments regarding PPS were found to be neutral. These results also suggest that patients are open to considering both the benefits and risks of PPS, which can lead to informed and balanced decision-making regarding their IC/BPS treatment. Both the breadth of discussion topics and sentiment demonstrate the complexity inherent in individual consideration of a medication with publicized side effects. Future studies of sentiment analyses toward other IC/BPS treatments and their comparison to PPS may provide additional context and direction in the treatment of this painful condition.

## Abbreviations

FDA: Food and Drug Administration
IC/BPS: interstitial cystitis/bladder pain syndrome
LDA: latent Dirichlet allocation
OR: odds ratio
PPS: pentosan polysulfate sodium
VADER: Valence Aware Dictionary for Sentiment Reasoning
